# Comparative buccopharyngeal morphology of tadpoles of Sphaenorhynchini (Anura: Hylidae: Hylinae)

**DOI:** 10.1007/s00114-025-02009-8

**Published:** 2025-08-21

**Authors:** Pedro Henrique dos Santos Dias, Barbara Caroline Marcondes, Yhasmynn Pensee Pinheiro Campos, Luiz Norberto Weber, Caio Vinicius de Mira-Mendes, Katyuscia Araujo-Vieira

**Affiliations:** 1 Senckenberg Biodiversity and Climate Research Centre (SBiK-F), Frankfurt Am Main, Germany; 2 Laboratório de Herpetologia, Departamento de Zoologia, Instituto de Ciências Biológicas, Universidade Federal de Minas Gerais, Belo Horizonte, Minas Gerais Brazil; 3 Departamento de Biologia, Universidade Estadual Do Maranhão, São Luís, Maranhão, Brazil; 4 Centro de Formação em Ciências Ambientais, Universidade Federal do Sul da Bahia, Porto Seguro-Eunápolis, Bahia, Brazil; 5 División Herpetología, Museo Argentino de Ciencias Naturales “Bernardino Rivadavia”-CONICET, Buenos Aires, Argentina

**Keywords:** Lime treefrogs, Internal oral anatomy, Synapomorphy, Systematics

## Abstract

The hylid tribe Sphaenorhynchini includes 15 small, greenish treefrog species within the genera *Sphaenorhynchus* and *Gabohyla*. *Sphaenorhynchus* is divided into three species groups: *S. lacteus*, *S. planicola*, and *S. platycephalus*, with *S. carneus* and *S. prasinus* remaining unassigned to any group; *Gabohyla* is monotypic. Larvae of most species have been described, except for *S. botocudo*, *S. cammaeus*, and *S. mirim*. Although larval morphology holds phylogenetic and diagnostic importance in Sphaenorhynchini, internal morphology remains largely unknown, with only the presence of a lingual papilla noted in *S. dorisae* and *S. lacteus*. To fill this gap, we describe the buccopharyngeal cavity of *G. pauloalvini*, *S. prasinus*, *S. dorisae*, *S. lacteus*, *S. canga*, and *S. palustris*. We provide a detailed description of this system for the tribe and discuss putative new larval synapomorphies. Tadpoles of Sphaenorhynchini show similarities in buccopharyngeal cavity morphology but vary in features such as prenarial arena shape, lateral ridge papillae, median ridge, and postnarial crest development. *Gabohyla pauloalvini* shows reduced lateral ridge papillae, which is an autapomorphy. Lateral roof papillae and a second pair of lateral ridge papillae are putative synapomorphies of the *S. platycephalus* group or one of its internal clades. The postnarial crest, present in all species, may be a synapomorphy of Sphaenorhynchini. These results enhance understanding of larval morphology and its systematic relevance within the tribe.

**Supplementary Information:**

The online version contains supplementary material available at 10.1007/s00114-025-02009-8.

## Introduction

The hylid tribe Sphaenorhynchini currently comprises 15 species of small greenish treefrogs from the genera *Sphaenorhynchus* (Tschudi 1838) and *Gabohyla* (Araujo-Vieira et al. 2020). *Sphaenorhynchus* is divided into three species groups: the *S. lacteus*, *S. planicola*, and *S. platycephalus* groups, while *S. carneus* and *S. prasinus* remain unassigned to any group (Araujo-Vieira et al. 2019). Meanwhile, *Gabohyla* is a monotypic genus, with *G. pauloalvini* as the only species described (Araujo-Vieira et al. 2020).

The larvae of most Sphaenorhynchini species have been described; however, those of *Sphaenorhynchus botocudo*, *S. cammaeus*, and *S. mirim* remain unknown (Caramaschi et al. 2009; Roberto et al. 2017). External morphological characters of the larvae, such as anteriorly directed nostrils with fleshy rim projections and a medial vent tube with dark pigmentation on its ventral surface, optimized as synapomorphies of Sphaenorhynchini, while the enlarged marginal papillae in the larval oral discs is a synapomorphy of *Sphaenorhynchus* (Araujo-Vieira et al. 2019, 2020). In addition to their phylogenetic significance, larval morphological traits are essential diagnostic characteristics for distinguishing species within the tribe. For instance, a short spiracle distinguishes larvae of *S. caramaschii* from those of *S. palustris* (which has an extremely long spiracle), *S. bromelicola*, *S. platycephalus*, and *S. surdus* (which have medium-sized spiracles), among others (Bokermann 1973; Cruz and Peixoto 1980; Nunes et al. 2007; Caramaschi 2010; Araujo-Vieira et al. 2015a, b).

Despite the significance of larval characters in Sphaenorhynchini, other than external morphology, the only known information regarding their internal morphology is the presence of a lingual papilla in *Sphaenorhynchus dorisae* and *S. lacteus* (Dias et al. 2023). To fill this gap, we describe here the buccopharyngeal cavity of *Gabohyla pauloalvini*, *S. prasinus* (unassigned group), *S. dorisae*, *S. lacteus* (the *S. lacteus* group), *S. canga*, and *S. palustris* (the *S. platycephalus* group), providing a detailed description of this system for Sphaenorhynchini. Additionally, we discuss possible new larval synapomorphies for the tribe, its genera, and internal clades.

## Materials and methods

### Buccopharyngeal morphology assessment

The examined tadpoles are housed in the herpetological collections of the Coleção de Zoologia, Universidade Federal do Sul da Bahia (CZUFSB), Instituto de Ciencias Naturales, Universidad Nacional de Colombia, Bogotá (ICN), Museu de Zoologia, Universidade Estadual de Santa Cruz (MZUESC), and Coleção de Girinos do Centro de Coleções Taxonômicas da Universidade Federal de Minas Gerais, Belo Horizonte (UFMG-GIR). *Sphaenorhynchus dorisae* (ICN 45612) and *S. lacteus* (ICN 45627) are from Letícia, Amazonas, Colombia. *Gabohyla pauloalvini* (MZUESC 22093) and *S. prasinus* (MZUESC 22094, UFMG-GIR 1619) are from the campus of the Universidade Estadual de Santa Cruz (UESC), Ilhéus, Bahia, and Carlos Chagas, Minas Gerais, Brazil, respectively. *Sphaenorhynchus canga* (UFMG-GIR 665) is from Chapada de Canga, Mariana, Minas Gerais, Brazil (type locality; Araujo et al. 2015a), and *S. palustris* (CZUFSB 528) is from the Reserva Particular do Patrimônio Natural (RPPN) Estação Veracel, Porto Seguro, Bahia, Brazil. Other examined species are listed in the Appendix.

We used the same larvae of *Gabohyla pauloalvini*, *Sphaenorhynchus canga*, *S. dorisae*, *S. lacteus*, and *S. prasinus* that were employed in the original descriptions or redescriptions of tadpoles by Suárez-Mayorga and Lynch (2001), Araujo-Vieira et al. (2015a), and Silva Neto et al. (2022). For larvae of *S. prasinus* from Carlos Chagas (UFMG-GIR 1619), we compared them with larvae from UESC (MZUESC 22094) redescribed by Silva Neto et al. (2022). For *S. palustris*, species assignment was done by comparing the larvae with the original description through diagnostic characters. One key diagnostic character is the presence of an extremely long spiracle, which distinguishes larvae of *S. palustris* from all those of Sphaenorhynchini that have been described (which have short and medium-sized spiracles; see Nunes et al. 2007; Araujo-Vieira et al. 2015b, 2019). Additionally, the tadpoles analyzed are from the same locality as those in the original description by Nunes et al. (2007).

At least one tadpole per species was dissected using the guidelines of Wassersug (1976); tadpoles of *Sphaenorhynchus dorisae* and *S. lacteus* were also submitted to the protocol for scanning electron microscopy (SEM) of Dias and Anganoy-Criollo (2024). The terminology of buccopharyngeal morphology is that of Wassersug (1976, 1980); the developmental stages are those of Gosner (1960).

### Taxonomy, phylogenetics, and character optimization

We follow the species group delimitation and taxonomy of Sphaenorhynchini proposed by Araujo-Vieira et al. (2019, 2020) and consider *Gabohyla* the valid generic name for *pauloalvini*.

The phylogenetic position of Sphaenorhynchini within Hylinae is controversial, and different authors have recovered it as the sister taxon to Scinaxini (Frost et al. 2006; Pyron and Wiens 2011; Duellman et al. 2016; Faivovich et al. 2018; Jetz and Pyron 2018; Portik et al. 2023), as sister to Dendropsophini (Faivovich et al. 2005; Araujo-Vieira et al. 2019; Orrico et al. 2021), or even as sister to Lophyohylini (Araujo-Vieira et al. 2023).

In light of this phylogenetic uncertainty, we conducted a review of the literature on buccopharyngeal morphology within Hylidae to understand how some characters evolved within Sphaenorhynchini, based on the data provided by Dias et al. (2024), and supplemented with recent publications (e.g., Marcondes et al. 2025; see Table S1). We proposed four transformation series (Hennig 1966; Grant and Kluge 2004) to account for the observed variation within Sphaenorhynchini. The phenotypic data matrix includes four characters and 80 terminals (scored based on data available until April 2025; see Appendix S1) edited using Mesquite v.3.70 (Maddison and Maddison 2021). We performed parsimony optimizations (Fitch 1971) of these characters on the most recent comprehensive phylogenetic hypothesis for Hylidae (Portik et al. 2023) using T.N.T. v1.6 (Goloboff and Morales 2023). The single multistate character was treated as nonadditive.

## Results

### Buccopharyngeal cavity

#### *Gabohyla pauloalvini*

**Fig. 1:**
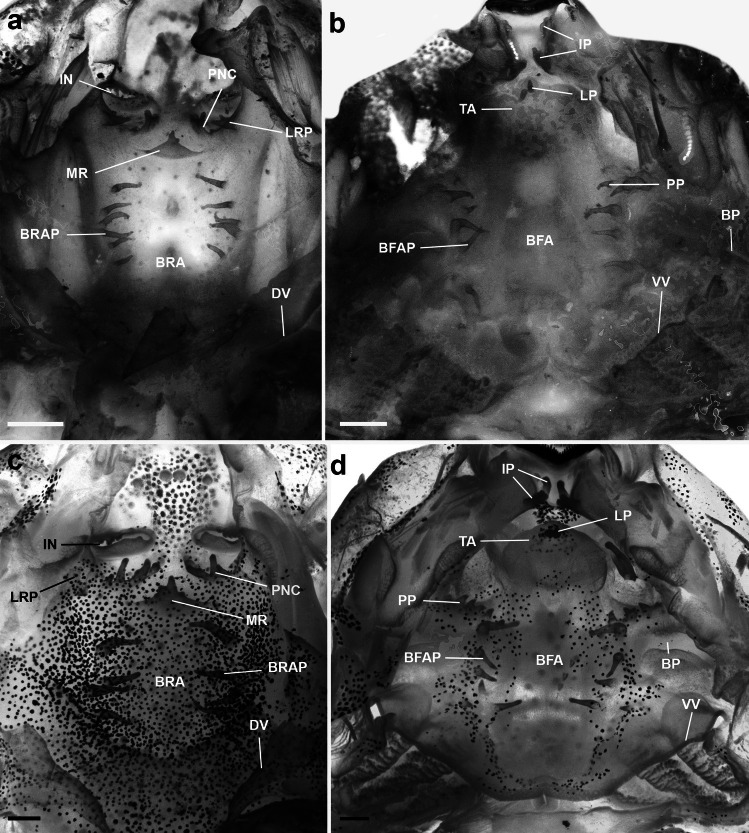
Buccopharyngeal cavity. Buccal roof and buccal floor of **a**, **b** *Gabohyla pauloalvini* (MZUESC 22093) at stage 36 and **c**, **d** *Sphaenorhynchus prasinus* (UFMG-GIR 1619) at stage 33. *Abbreviations*: BFA, buccal floor arena; BFAP, buccal floor arena papilla; BP, buccal pocket; BRA, buccal roof arena; BRAP, buccal roof arena papilla; DV, dorsal velum; IN, internal nare; IP, infralabial papillae; LP, lingual papilla; LRP, lateral ridge papilla; MR, median ridge; PNC, postnarial crest; PP, prepocket papilla; TA, tongue anlage; VV, ventral velum. Scale bars = 1 mm (**a**, **b**) and 2 mm (**c**, **d**). The LRP on the right side was accidentally damaged during dissection of *S. prasinus* in **c**

Buccal roof (Fig. 1a) triangular. Prenarial arena elliptical, with few pustulations. Internal nares elliptical, transversally oriented; anterior margin with two triangular projections; posterior valve free, with a small triangular projection. Vacuities absent. Postnarial arena diamond-shaped, with a small longitudinal dermal crest with three projections (one smaller, and two conical), oriented at an angle of 45°. Lateral ridge papillae present, small, conical, and bifurcated. Median ridge tall, wide, and triangular, with a small central projection. Buccal roof arena delimited by five to six conical papillae on each side; buccal roof papillae absent. Few scattered pustulations on the buccal roof arena. Glandular zone well defined, with marked secretory pits. Dorsal velum V-shaped, medially interrupted, with marginal projections.

Buccal floor (Fig. 1b) triangular. Two pairs of infralabial papillae; first pair (medial) short, globose; second pair (lateral) semi-triangular. Tongue anlage well developed, rounded, bearing a single pair of short, conical lingual papillae. Buccal floor arena bell-shaped; four to five conical papillae on each side. Buccal floor arena with a few scattered pustulations. Two conical prepocket papillae. Buccal pockets deep, wide, and oblique slit-shaped. Ventral velum present; spicular support conspicuous; medial notch present; secretory pits well developed; secretory ridges present. Branchial basket triangular, wider than long. Glottis visible, posterior to the medial notch of the velum.

#### *Sphaenorhynchus prasinus*

Buccal roof (Fig. 1c) triangular. Prenarial arena triangular, with six pustulations transversely oriented. Internal nares elliptical, transversally oriented; anterior margin with three triangular projections; posterior valve with a small triangular projection. Vacuities absent. Postnarial arena diamond-shaped, with a long longitudinal dermal crest with four conical projections, oriented at an angle of 45°. Lateral ridge papillae present, well developed, bifurcated. Median ridge tall, triangular, with small triangular projections. Buccal roof arena delimited by four tall, conical papillae on each side; buccal roof papillae absent. Few scattered pustulations on the buccal roof arena. Glandular zone well defined, with marked secretory pits. Dorsal velum V-shaped, medially interrupted, devoid of papillae or projections.

Buccal floor (Fig. 1d) triangular. Two pairs of infralabial papillae; first pair (medial) finger-like, second pair (lateral) hand-like. Tongue anlage well-developed, bearing a single pair of tall, conical lingual papillae. Buccal floor arena bell-shaped; five to six conical papillae on each side. Buccal floor arena with a few scattered pustulations. Four small prepocket papillae present. Buccal pockets deep, wide, and oblique slit-shaped. Ventral velum present; spicular support not visible; small medial notch present; secretory pits well-developed; secretory ridges present. Branchial basket triangular, wider than long. Glottis visible, posterior to the medial notch of the velum.

### The *Sphaenorhynchus lacteus* group

#### *Sphaenorhynchus dorisae*

**Fig. 2:**
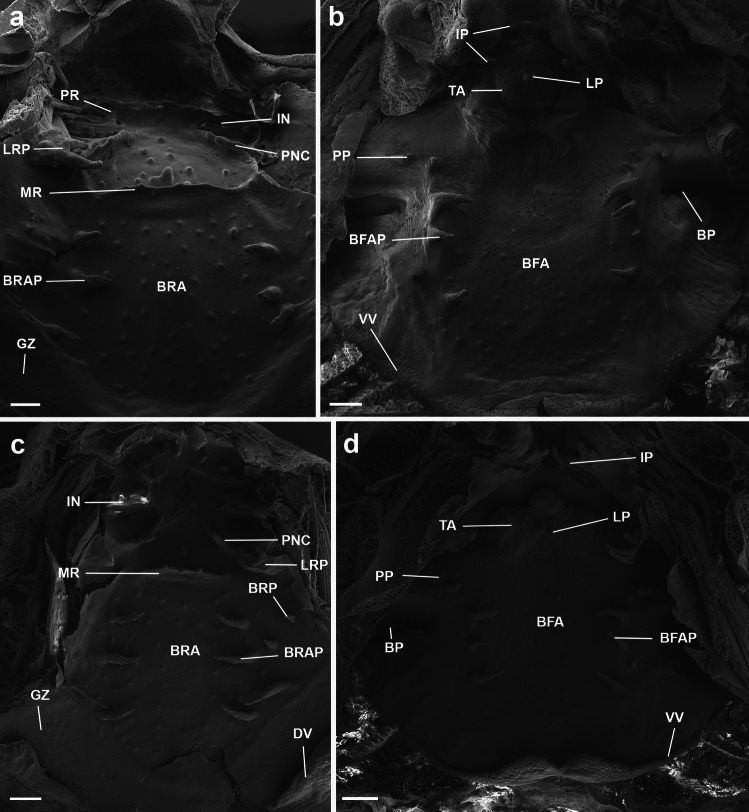
Buccopharyngeal cavity of the tadpoles of the *Sphaenorhynchus lacteus* group. Buccal roof and buccal floor of **a**, **b** *Sphaenorhynchus dorisae* (ICN 45612) at stage 34 and **c**, **d** *S. lacteus* (ICN 45627) at stage 35. *Abbreviations*: BFA, buccal floor arena; BFAP, buccal floor arena papilla; BP, buccal pocket; BRA, buccal roof arena; BRAP, buccal roof arena papilla; BRP, buccal roof papilla; DV, dorsal velum; GZ, glandular zone; IN, internal nare; IP, infralabial papillae; LP, lingual papilla; LRP, lateral ridge papilla; MR, median ridge; PNC, postnarial crest; PR, postnarial projection; PP, prepocket papilla; TA, tongue anlage; VV, ventral velum. Scale bars = 200 μm

Buccal roof (Fig. 2a) triangular. Prenarial arena triangular, devoid of pustulations or papillae. Internal nares elliptical, transversally oriented; posterior valve free, with a tall, triangular postnarial projection. Vacuities absent. Postnarial arena diamond-shaped, with a long, longitudinal dermal crest, with two or three terminal projections, oriented at an angle of 45°. Lateral ridge papillae present, well-developed, trifurcated. Median ridge low, wide, and trapezoidal, without projections. Buccal roof arena delimited by three conical papillae on each side; buccal roof papillae absent. Few scattered pustulations on the buccal roof arena. Glandular zone well defined, with marked secretory pits. Dorsal velum V-shaped, medially interrupted, devoid of papillae or projections.

Buccal floor (Fig. 2b) triangular. Two pairs of globose, infralabial papillae; first pair (medial) short, finger-like, second pair (lateral) semi-triangular. Tongue anlage well-developed, rounded, bearing a single pair of short, conical lingual papillae. Buccal floor arena bell-shaped; five to six finger-like papillae on each side. Buccal floor arena with a few scattered pustulations. Single prepocket papilla present. Buccal pockets deep, wide, and oblique slit-shaped. Ventral velum present; spicular support conspicuous; medial notch present; secretory pits well-developed; secretory ridges present. Branchial basket triangular, wider than long. Glottis visible, posterior to the medial notch of the velum.

#### *Sphaenorhynchus lacteus*

Buccal roof (Fig. 2c) triangular. Prenarial arena triangular, with four pustulations. Internal nares elliptical, transversally oriented; anterior margin with three triangular projections; posterior marginal valve free. Vacuities absent. Postnarial arena diamond-shaped, with a long, longitudinal dermal crest, with a small central, terminal projection, oriented at an angle of 45°. Lateral ridge papillae present, well-developed, bifurcated. Median ridge tall, triangular, with small triangular projections. Buccal roof arena delimited by four to five tall, conical papillae on each side; 2–3 buccal roof papillae present. A few pustulations scattered on the buccal roof arena. Glandular zone well defined, with marked secretory pits. Dorsal velum V-shaped, medially interrupted, devoid of papillae or projections.

Buccal floor (Fig. 2d) triangular. Two pairs of infralabial papillae; first pair (medial) finger-like, second pair (lateral) bilobed. Tongue anlage well developed, bearing a single pair of tall, finger-like lingual papillae. Buccal floor arena bell-shaped; three conical papillae on each side. Buccal floor arena with a few scattered pustulations. Two prepocket papillae present. Buccal pockets deep, wide, oblique slit shaped. Ventral velum present; spicular support conspicuous; medial notch present; secretory pits well developed; secretory ridges present. Branchial basket triangular, wider than long. Glottis visible, posterior to the medial notch of the velum.

### The *Sphaenorhynchus platycephalus* group

#### *Sphaenorhynchus canga*

**Fig. 3:**
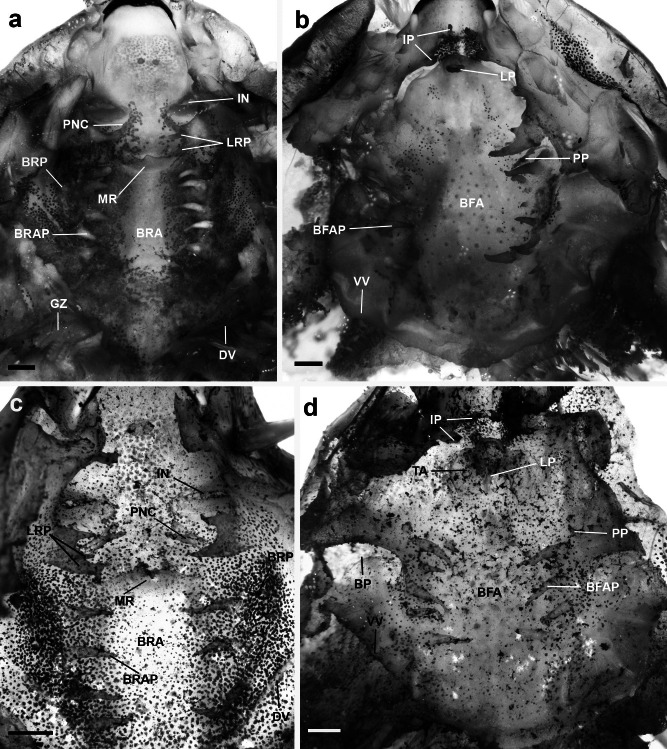
Buccopharyngeal cavity of the tadpoles of the *Sphaenorhynchus platycephalus* group. Buccal roof and buccal floor of **a**, **b** *Sphaenorhynchus canga* (UFMG- GIR 665) at stage 36 and **c**,** d** *S. palustris* (CZUFSB 528) at stage 32. *Abbreviations*: BFA, buccal floor arena; BFAP, buccal floor arena papilla; BP, buccal pocket; BRA, buccal roof arena; BRAP, buccal roof arena papilla; BRP, buccal roof papilla; DV, dorsal velum; GZ, glandular zone; IN, internal nare; IP, infralabial papillae; LP, lingual papilla; LRP, lateral ridge papilla; MR, median ridge; PNC, postnarial crest; PP, prepocket papilla; TA, tongue anlage; VV, ventral velum. Scale bar = 2 mm (**a**, **b**) and 1 mm (**c**, **d**)

**Fig. 4:**
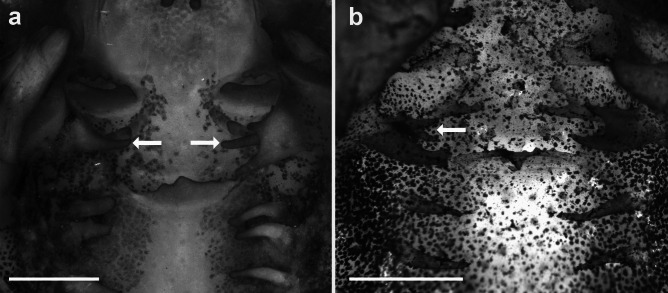
Detail of the two pairs of lateral ridge papillae of the buccal roof of the tadpoles of the *Sphaenorhynchus platycephalus* group. **a** *Sphaenorhynchus canga* (UFMG- GIR 665) at stage 36 and **b** *S. palustris* (CZUFSB 528) at stage 32. White arrows indicate the second pair of lateral ridge papillae (LRP). The first pair of LRP was completely and partially removed on the right side in **a** and **b**, respectively. Scale bars = 1 mm

Buccal roof (Fig. 3a) triangular. Prenarial arena triangular, with two short, rounded, simple papillae. Internal nares elliptical, transversally oriented; anterior margin with 2–4 triangular projections; posterior marginal valve free, with a tall, triangular projection. Vacuities absent. Postnarial arena diamond-shaped, with a long, longitudinal dermal crest, with three or four terminal projections, oriented at an angle of 45°. Postnarial papillae absent. Two pairs of lateral ridge papillae, first pair well developed, bifurcated; second pair tall, conical (Fig. 4a). Median ridge low, wide, and trapezoidal, with two small projections. Buccal roof arena delimited by five tall, conical papillae on each side; 2–3 buccal roof papillae present. Few scattered pustulations on the buccal roof arena. Glandular zone well defined, with marked secretory pits. Dorsal velum V-shaped, medially interrupted, devoid of papillae or projections.

Buccal floor (Fig. 3b) triangular. Two pairs of infralabial papillae; first pair (medial) tall, conical; second pair (medial) short, globose. Tongue anlage well-developed, rounded, bearing a single pair of tall, conical lingual papillae. Buccal floor arena bell-shaped; five to six conical papillae on each side. Buccal floor arena with a few scattered pustulations. Two or three conical prepocket papillae. Buccal pockets deep, wide, and oblique slit-shaped. Ventral velum present; spicular support conspicuous; medial notch absent; secretory pits well-developed; secretory ridges present. Branchial basket triangular, wider than long. Glottis visible, posterior to the medial notch of the velum.

#### *Sphaenorhynchus palustris*

Buccal roof (Fig. 3c) triangular. Prenarial arena triangular, with single rounded, bilobed papillae. Internal nares elliptical, transversally oriented; anterior margin with two triangular projections; posterior marginal valve free, with a tall, medial triangular projection. Vacuities absent. Postnarial arena diamond-shaped, with a long, longitudinal dermal crest, with two terminal projections, oriented at an angle of 45°. Postnarial papillae absent. Two pairs of lateral ridge papillae, first pair well developed, bifurcated; second pair short, bifurcated (Fig. 4b). Median ridge low, wide, and triangular, with three small terminal projections. Buccal roof arena delimited by three to four tall, conical papillae on each side; 2–3 buccal roof papillae present. Few scattered pustulations on the buccal roof arena. Glandular zone well defined, with marked secretory pits. Dorsal velum V-shaped, medially interrupted, devoid of papillae or projections.

Buccal floor (Fig. 3d) triangular. Two pairs of infralabial papillae; first pair finger-like, second pair cross-shaped. Tongue anlage well developed, rounded, bearing a single pair of tall, conical lingual papillae. Buccal floor arena bell-shaped; five to six tall, conical papillae on each side. Buccal floor arena with a few scattered pustulations. Three to four conical prepocket papillae present. Buccal pockets deep, wide, and oblique slit-shaped. Ventral velum present; spicular support conspicuous; medial notch present. Secretory pits well developed; secretory ridges present. Branchial basket triangular, wider than long. Glottis visible, posterior to the medial notch of the velum.

### Comparisons

Sphaenorhynchini tadpoles exhibit a generally similar buccopharyngeal cavity morphology. In the buccal roof, the main differences are in the prenarial arena, postnarial papillae, lateral ridge papillae, and median ridge. *Gabohyla pauloalvini* has an elliptical prenarial arena with few pustulations, while all *Sphaenorhynchus* species have a triangular prenarial arena, varying in the presence (absent in *S. dorisae*) and number of pustulations (five in *S. lacteus* and six in *S. prasinus*) and papillae (two in *S. canga* and one in *S. palustris*); prenarial papillae are bilobed in *S. palustris* and simple in *S. canga*. All *Sphaenorhynchus* species have well-developed, bi- or trifurcate first pair of lateral ridge papillae, whereas in *G. pauloalvini*, they are reduced and smaller than the median ridge.

A second pair of lateral ridge papillae is present in* Sphaenorhynchus canga* (tall and conical) and *S. palustris* (short and bifurcated). The median ridge also varies in shape and projections, with *Gabohyla pauloalvini*, *S. lacteus*, and *S. prasinus* having tall, triangular ridges with small projections; *S. canga* and *S. dorisae* having lower, wider, and trapezoidal ridges with small projections (projections absent in *S. dorisae*); while *S. palustris* has low, wide, and triangular ridges with three terminal projections. Additionally, the buccal roof arena is well-defined in *G. pauloalvini* and *S. canga* but poorly delimited in *S. dorisae*, *S. lacteus*, and *S. prasinus*, differing in the number (two to four papillae in *S. dorisae*, *S. lacteus*, *S. palustris*, and *S. prasinus*) of buccal roof arena papillae (five to six papillae in *G. pauloalvini* and *S. canga*). Buccal roof papillae are present in *S. canga*, *S. lacteus*, and *S. palustris* (absent in *G. pauloalvini*, *S. dorisae*, and *S. prasinus*).

We also observed variations in the development and shape of the postnarial crest in the buccal roof. *Gabohyla pauloalvini* has a smaller dermal crest with three projections (one smaller and two conical) than those of *Sphaenorhynchus* species, which show projections varying in both number (one in *S. lacteus* and two to four in the other species) and shape, with terminal (*S. canga*, *S. dorisae*, *S. lacteus*, *S. palustris*) and conical (*S. prasinus*) forms.

The buccal floor also shows distinct variations across species, particularly in the infralabial papillae, prepocket papillae, and the ventral velum. *Gabohyla pauloalvini*, *Sphaenorhynchus canga*, and *S. dorisae* have globose, semi-triangular, or conical infralabial papillae, while *S. lacteus*, *S. palustris*, and *S. prasinus* possess more complex shapes, including bilobed, cross-shaped, or hand-like structures. The number of prepocket papillae is another distinguishing characteristic, with *G. pauloalvini* and *S. lacteus* having two, *S. dorisae* a single one, *S. canga* and *S. palustris* two or three, and *S. prasinus* four. The ventral velum is present in all species, but *S. prasinus* lacks visible spicular support, unlike the others. Moreover, *S. canga* is the only species without a medial notch in the ventral velum.

### Character evolution

#### Character 1. Postnarial papillae, nature.

(0) Free, individual papillae; (1) fused into a dermal crest.

Postnarial papillae are located immediately behind the internal nares and are typically tall, conical structures (Wassersug 1976, 1980). However, they may alter in shape, as seen in some funnel-mouth tadpoles (e.g., Dias et al. 2021a). In all examined Sphaenorhynchini, a dermal crest is found at the same topological location as the postnarial papillae, which we hypothesized has evolved from the fusion of these papillae.

**Fig. 5:**
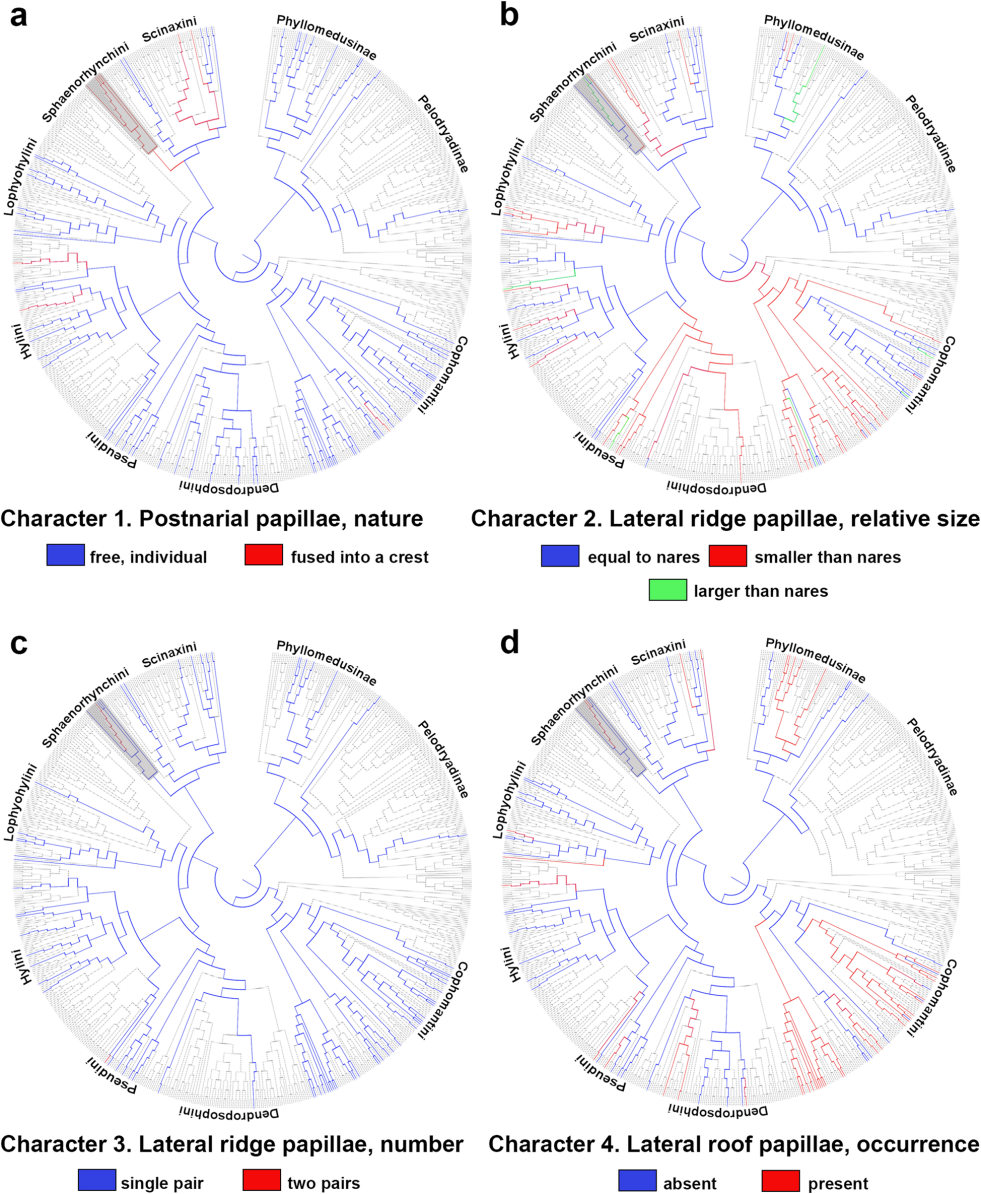
Parsimonious optimization of characters **a** 1, **b** 2, **c** 3, and **d** 4 for Hylidae mapped onto the phylogenetic tree of Portik et al. (2023). Light gray indicates unknown character states. Clade names represent their approximate positions in the tree. The gray rectangle highlights the Sphaenorhynchini tribe. For a more detailed view of the optimizations, see Appendix S1

Taxonomic distribution and optimization: the presence of a dermal crest is a putative synapomorphy of Sphaenorhynchini (Fig. 5a). We also noted this crest in *Scinax acuminatus* and *Scinax nasicus*, as well as in *Boana wavrini*, and a few Hylini.

#### Character 2. Lateral ridge papillae, relative size.

(0) Approximately equal to internal nares width, (1) smaller than internal nares width, (2) larger than internal nares width. Nonadditive.

The lateral ridge papillae can vary in size, shape, and overall morphology. Within our sampled taxa, we observed variation in the size of this feature in relation to the width of the internal nares. To measure the size of the internal nares, we disregarded the area of the narial vacuities present in several hylids, particularly in Cophomantini (e.g., Kolenc et al. 2008).

Taxonomic distribution and optimization: a reduced lateral ridge papilla is a putative autapomorphy of *Gabohyla pauloalvini* (Fig. 5b). There are multiple transformations between states within Hylidae. Current distribution of the data also seems to suggest that a reduced lateral ridge would be a synapomorphy for Cophomantini and Pseudini + Dendropsophini, pending further observations.

#### Character 3. Lateral ridge papillae, number.

(0) Single pair, (1) two pairs.

Taxonomic distribution and optimization: a second pair of lateral ridge papillae was only observed in *Sphaenorhynchus canga* and *S. palustris* (the latter was not included in Portik et al. 2023; Fig. 5c).

#### Character 4. Lateral roof papillae, occurrence.

(0) Absent, (1) present.

Lateral roof papillae are (usually) conical papillae scattered at the lateral portion of the buccal roof, lateral to the buccal roof arena papillae (Wassersug 1976, 1980).

Taxonomic distribution and optimization: the lateral roof papillae evolved independently in several lineages (Fig. 5d). They are present in *Sphaenorhynchus canga*, *S. palustris* (not included in Portik et al. 2023), and *S. lacteus*, suggesting that further sampling within *Sphaenorhynchus* is necessary to better test the evolution of this character within the tribe. Regarding other hylids, they are present in many Cophomantini and could be a putative synapomorphy for *Pithecopus* + *Phyllomedusa*, pending further analysis.

## Discussion

### Larval morphology and systematics

**Fig. 6:**
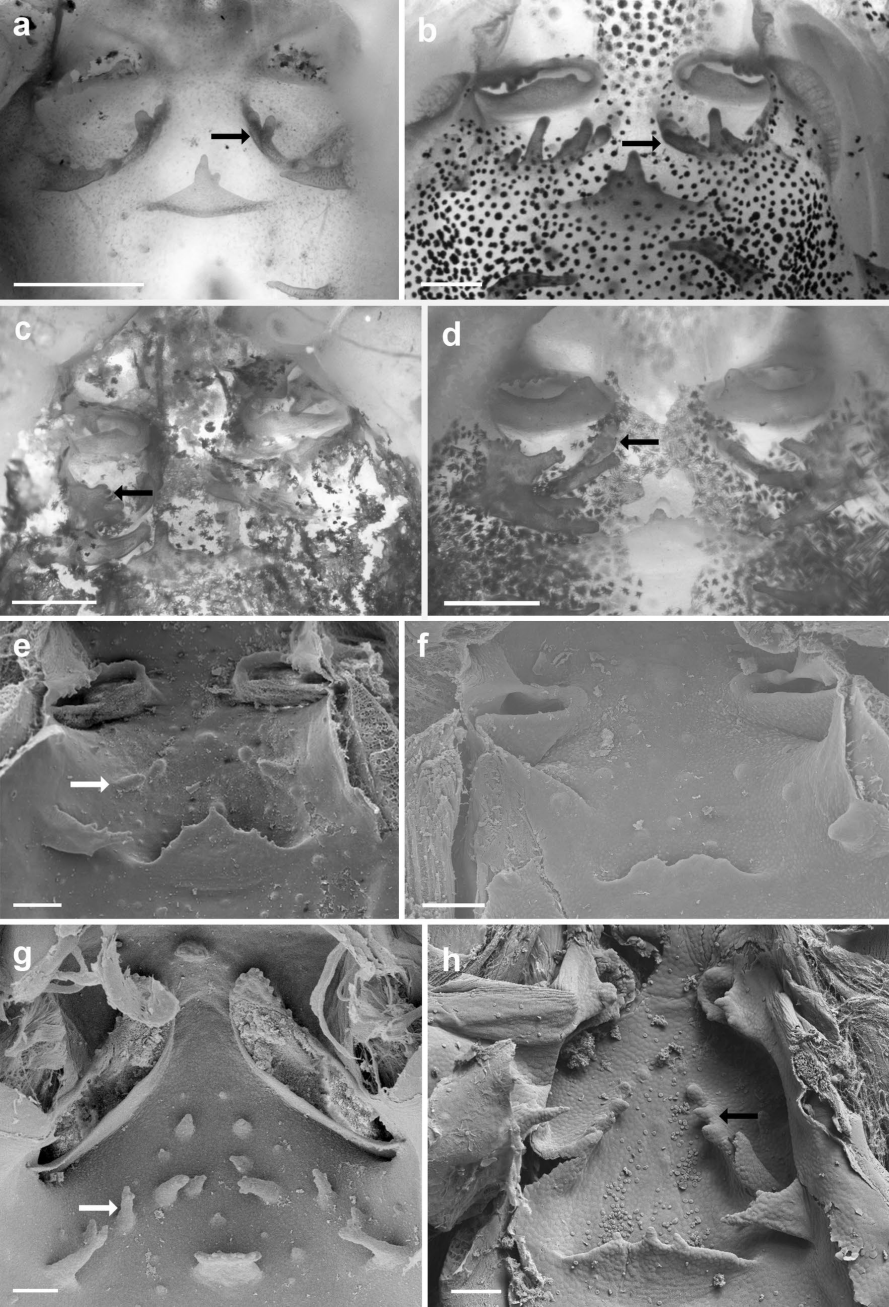
Detail of the postnarial arena of the buccal roof of **a** *Gabohyla pauloalvini*, **b** *Sphaenorhynchus prasinus*, **c** *S. lacteus*, **d** *S. canga*, **e** *Dendropsophus molitor*, **f** *Xenohyla truncata*, **g** *Ololygon albicans*, and **h ***Thoropa megatympanum*. Black arrow indicating postnarial crest (**a**–**d**, **h**); postnarial papilla and crest absent in **f**; white arrow indicating postnarial papilla (**e**, **g**). Scale bars = 100 μm

The buccopharyngeal cavity of *Gabohyla* and *Sphaenorhynchus* tadpoles is quite similar to that of many hylid species (e.g., Wassersug 1980; Vera Candioti 2007; Alcalde et al. 2011; Dias 2023; Dias et al. 2019, 2023). The most distinctive feature is the presence of a postnarial crest in the buccal roof of all examined species. This crest is found in the postnarial arena, the same region typically occupied by the postnarial papillae (Wassersug 1976, 1980). We hypothesize that in Sphaenorhynchini, it evolved through the fusion of the postnarial papillae (Fig. 6a–d).

This crest is absent (Fig. 6e–h) in the larvae of *Dendropsophus* (e.g., *D. cerradensis*, *D. decipiens*, *D. ebraccatus*, *D. molitor*, *D. minutus*, *D. microcephalus*, *D. soaresi*; Wassersug 1980; Echeverría 1997; Kaplan and Ruíz 1997; Vera Candioti 2007; Dias et al. 2019; Costa et al. 2024; Marcondes et al. 2025; Fig. 6e), *Xenohyla truncata* (Dias et al. 2023; Fig. 4f), and in most Scinaxini (e.g., Conte et al. 2007; Vera Candioti 2007; Alcalde et al. 2011; Dias and Pie 2021), although it is present in *Scinax acuminatus* and *S. nasicus* (Sandoval 2000). A similar condition has only been described in the semi-terrestrial tadpoles of Cycloramphidae (Dias et al. 2021b; Fig. 6h) and in the distantly related hylids, such as the adherent larvae of *Boana semilineata* (D’Heursel and de Sá 1999), *Duellmanohyla schmidtorum*, *Ptychohyla leonhardschultzei* (Wassersug 1980), and *Hyla plicata* (Kaplan and Ramírez-Bautista 1996). Given the distribution of this character within Hylidae, the presence of the postnarial crest may represent a synapomorphy of Sphaenorhynchini.

Given the presence of the postnarial crest in various ecomorphological types of tadpoles, including semiterrestrial, adherent, and nektonic, speculating about its function is challenging. Few authors have discussed the function of the postnarial papillae. For instance, Kenny (1969a, b) referred to them as “sensitive.” Typically, these papillae are tall, conical, and found in two or three pairs (e.g., Wassersug 1980). They are absent in several taxa, particularly in endotrophic (e.g., Dubeux et al. 2023) and macrophagous (e.g., Vera Candioti et al. 2021) larvae. Some ecomorphs, such as funnel-mouthed tadpoles, exhibit modified forms often characterized by an enlarged, obtuse papilla (e.g., Wassersug 1980; Dias et al. 2018, 2021b). Wassersug (1980) noted that the postnarial arena aligns perfectly with the tongue anlage, which could imply a gustatory function; additionally, these structures may play a mechanical role in sorting particles that enter the buccopharyngeal cavity or in directing the flow of water. Further studies are needed to clarify the function of these and other elements within the buccopharyngeal cavity.

Another interesting characteristic is the reduced lateral ridge papillae in *Gabohyla*. All *Sphaenorhynchus* have a regular (approximately equal to the internal nares width) or large (larger than the internal nares width), bi- or trifurcate lateral ridge papilla, while in *G. pauloalvini*, it is reduced and smaller than the internal nares. Wassersug (1980:114) stated that the lateral ridge papillae have a direct relation to the postnarial and roof arenas, but this has never been tested. The reduced lateral ridge in *G. pauloalvini* contradicts that hypothesis, as both postnarial and roof arenas are present and not reduced. Similarly, in *S. canga* and *S. prasinus*, there is no evident increase in the size or development of these areas, and *S. canga* even has a poorly delimited buccal roof arena.

The relative size of the lateral ridge papillae within hylids is quite variable, with instances of reduction in all Hylinae tribes. The lateral ridge papillae are reduced in some *Ololygon* (e.g., *O. v-signata*; Dias and Pie 2021), in most *Trachycephalus* (e.g., Provete et al. 2021), in some *Hyla* (Viertel 1982), in most Dendrosophini (e.g., Dias et al. 2019, 2023), and in several Cophomantini (e.g., D’Heursel and Haddad 2007). In Sphaenorhynchini, the reduction of the lateral ridge papillae is a putative autapomorphy of *Gabohyla*.

The presence of a second pair of lateral ridge papillae in *Sphaenorhynchus canga* and *S. palustris*—tall and conical in the former, short and bifurcated in the latter—suggests a possible synapomorphy for the *S. platycephalus* species group or for a more restricted internal clade, as these species belong to sister clades within the group (Araujo-Vieira et al. 2019). The occurrence of a second or more pairs of lateral ridge papillae is rare in Hylidae; in all species included in our data matrix, it is polymorphic in *Pseudacris regila*, which may present a second pair in some specimens (Wassersug 1976). A second pair of lateral ridge papillae was also described for *Bromeliohyla dendroscarta* and *Duellmanohyla schmidtorum* (Wassersug 1980), but these species were not included in the sampling of Portik et al. (2023). Outside of Hylidae, the second pair of lateral ridge papillae has been reported in Alsodidae (two pairs in *Alsodes* sp.) and Leptodactylidae (two pairs in *Crossodactylodes* sp., and two fused pairs in *Physalaemus jordanensis*; Wassersug and Heyer 1988; Gomes et al. 2010).

Similarly, we observed lateral roof papillae in tadpoles of *Sphaenorhynchus canga*, *S. palustris*, and *S. lacteus*, suggesting that the presence of these papillae could be another putative synapomorphy for the *S. platycephalus* group or one of its internal clades, with independent occurrence in *S. lacteus*. However, to test both hypotheses, it is necessary to verify this condition at least in the larvae of *S. cammaeus* and *S. caramaschii* (Caramaschi 2010; Roberto et al. 2017), the two earlier divergent taxa of the *S. platycephalus* group (Araujo-Vieira et al. 2019). Our observations indicate that the lateral roof papillae evolved independently in several lineages of Hylidae, including two independent evolutions within Scinaxini, in *Julianus uruguayus* and *Scinax acuminatus* (Alcalde et al. 2011). Such papillae are also present in some taxa not included in Portik et al. (2023), such as *Ololygon aromothyella* (Alcalde et al. 2011), suggesting that their presence could be more widely distributed within that tribe.

## Conclusion

Our study provides detailed descriptions of the buccopharyngeal cavity of *Gabohyla pauloalvini*, *Sphaenorhynchus prasinus*, *S. dorisae*, *S. lacteus*, *S. canga*, and *S. palustris*, filling a gap in understanding Sphaenorhynchini larval morphology and contributing to a better understanding of their phylogenetic relationships. The presence of a postnarial crest in all examined Sphaenorhynchini species is a putative synapomorphy for the tribe. *Gabohyla pauloalvini*’s reduced lateral ridge papillae is an autapomorphy, which also distinguishes it from *Sphaenorhynchus* species. The occurrence of a second pair of lateral ridge papillae and lateral roof papillae (with an instance of homoplasy in *S. lacteus*) in *S. canga* and *S. palustris* suggests the possibility of two synapomorphies for the *S. platycephalus* group or its internal clades, which need further investigation for corroboration.

Taken together, these findings provide additional morphological evidence for the monophyly of the tribe Sphaenorhynchini and its internal clades, as well as for distinguishing *Gabohyla* from *Sphaenorhynchus*. Additionally, they reveal interesting patterns of larval evolution within Hylidae and pave the way for future research on larval evolution within the family.

## Supplementary Information

Below is the link to the electronic supplementary material.

Table S1 (DOCX 22.4 KB)

Appendix S1 (TNT 22.8 KB)

## Data Availability

No datasets were generated or analysed during the current study.

## Appendix. Examined material

Institutional Abbreviations follow Sabaj (2022). *Dendrosophus molitor*. Colombia: Villavicencio, ICN 2007. *Xenohyla truncata*. Brazil: Rio de Janeiro, Maricá, Restinga de Maricá, MZUESC 224471. *Ololygon albicans*. Brazil: Teresópolis, UNIRIO 2729. *Thoropa megatympanum*. Brazil: Santa do Riacho, UFMG 158.

## References

[CR1] Alcalde L, Vera Candioti MF, Kolenc F, Borteiro C, Baldo D (2011) Cranial anatomy of tadpoles of five species of *Scinax* (Hylidae, Hylinae). Zootaxa 2787:19–36

[CR2] Araujo-Vieira K, Lacerda JVA, Pezzuti TL, Leite FS, Assis CL, Cruz CAG (2015a) A new species of hatchet-faced treefrog *Sphaenorhynchus Tschudi* (Anura: Hylidae) from quadrilátero ferrífero, Minas Gerais, southeastern Brazil. Zootaxa 4059:96–114 10.11646/zootaxa.4059.1.5 26701555

[CR3] Araujo-Vieira K, Tacioli A, Faivovich J, Orrico VGD, Grant T (2015b) The tadpole of *Sphaenorhynchus caramaschii*, with comments on larval morphology of *Sphaenorhynchus* (Anura: Hylidae). Zootaxa 3904:270–282 10.11646/zootaxa.3904.2.6 25660784

[CR4] Araujo-Vieira K, Blotto BL, Caramaschi U, Haddad CFB, Faivovich J, Grant T (2019) A total evidence analysis of the phylogeny of hatchet-faced treefrogs (Anura: Hylidae: *Sphaenorhynchus*). Cladistics 35:469–486 34618945 10.1111/cla.12367

[CR5] Araujo-Vieira K, Luna MC, Caramaschi U, Haddad CFB (2020) A new genus of lime treefrogs (Anura: Hylidae: Sphaenorhynchini). Zool Anz 286:81–89

[CR6] Araujo-Vieira K, Lourenço ACC, Lacerda JVL, Lyra ML, Blotto BL, Ron SR, Faivovich J (2023) Treefrog diversity in the Neotropics: phylogenetic relationships of Scinaxini (Anura: Hylidae: Hylinae). S Am J Herpetol 27:1–143

[CR7] Bokermann WCA (1973) Duas novas espécies de *Sphaenorhynchus* da Bahia (Anura, Hylidae). Rev Bras Biol 33:589–594

[CR8] Caramaschi U (2010) Descrição do girino de *Sphaenorhynchus surdus* (Cochran, 1953) (Anura, Hylidae). Bol Mus Biol Mello Leitão, Nova Sér (Online) 27:67–74

[CR9] Caramaschi U, Almeida AP, Gasparini JL (2009) Description of two new species of *Sphaenorhynchus* (Anura, Hylidae) from the state of Espírito Santo, southeastern Brazil. Zootaxa 2115:34–46

[CR10] Conte CE, Nomura F, Rossa-Feres DC, D’Heursel A, Haddad CFB (2007) The tadpole of *Scinax catharinae* (Anura, Hylidae) with description of the oral morphology, and a review of tadpoles from *Scinax catharinae* group. Amphib-Reptil 28:177–192

[CR11] Costa CA, Santos AJS, Silva RA, Sena FP, Uchôa LR, Andrade EB (2024) Description of internal oral anatomy and chondrocranium of *Dendropsophus soaresi* tadpole (Anura: Hylidae) with comments on its external morphology. S Am J Herpetol 30:52–62

[CR12] Cruz CAG, Peixoto OL (1980) Notas sobre o girino de *Sphaenorhynchus orophilus* (Lutz & Lutz, 1938) (Amphibia, Anura, Hylidae). Rev Bras Biol 40:383–386

[CR13] D’Heursel A, de Sá RO (1999) Comparing the tadpoles of *Hyla geographica* and *Hyla semilineata*. J Herpetol 33:353–361

[CR14] D’Heursel A, Haddad CF (2007) Anatomy of the oral cavity of hylid larvae from the genera *Aplastodiscus*, *Bokermannohyla*, and *Hypsiboas* (Amphibia, Anura): description and systematic implications. J Herpetol 41:458–468

[CR15] Dias PHS (2023) First description of buccopharyngeal anatomy in Pelodryadinae larvae: morphological comparison and systematic implications (Anura: Hylidae: Pelodryadinae: *Litoria rubella* and *Ranoidea caerulea*). J Morphol 284:e21651 37856280 10.1002/jmor.21651

[CR16] Dias PHS, Anganoy-Criollo M (2024) Harlequin frog tadpoles—comparative buccopharyngeal morphology in the gastromyzophorous tadpoles of the genus *Atelopus* (Amphibia, Anura, Bufonidae), with discussion on the phylogenetic and evolutionary implication of characters. Sci Nat 111:3 10.1007/s00114-024-01889-6 PMC10803719 38252296

[CR17] Dias PHS, Pie MR (2021) Buccopharyngeal morphology of the tadpoles of *Scinax v-signatus*, with comments on larval characters of the *S. perpusillus* species group (Amphibia: Anura: Hylidae). Zootaxa 4964:195–200 10.11646/zootaxa.4964.1.12 33903537

[CR18] Dias PHS, Anganoy-Criollo M, Guayasamin JM, Grant T (2018) The tadpole of *Epipedobates darwinwallacei* Cisneros-Heredia and Yánez-Muñoz, 2011 (Dendrobatidae: Colostethinae), with new synapomorphies for *Epipedobates*. S Am J Herpetol 13:54–63

[CR19] Dias PHS, Araujo-Vieira K, Carvalho-e-Silva AMPT, Orrico VGD (2019) Larval anatomy of *Dendropsophus decipiens* (A Lutz, 1925 (Anura: Hylidae: Dendropsophini) with considerations to the larvae of this genus. PlosOne 17:e0219716 10.1371/journal.pone.0219716 PMC6623958 31295323

[CR20] Dias PHS, Anganoy-Criollo M, Rada M, Grant T (2021a) The tadpoles of the funnel-mouthed dendrobatids (Anura: Dendrobatidae: Colostethinae: *Silverstoneia*): external morphology, musculoskeletal anatomy, buccopharyngeal cavity, and new synapomorphies. J Zool Syst Evol Res 59:691–717

[CR21] Dias PHS, Vera Candioti F, Sabbag AF, Colaço G, Silva HR, Haddad CFB, Carvalho-e-Silva AMPT, Grant T (2021b) Life on the edge: tadpoles of Cycloramphidae (Amphibia; Anura), anatomy, systematics, functional morphology, and comments on the evolution of semiterrestrial tadpoles. J Zool Syst Evol Res 59:1297–1321

[CR22] Dias PHS, Marcondes BC, Pezzuti TL, Vera Candioti F, Araujo-Vieira K, Prodocimo MM, Haas A (2023) The missing piece of the puzzle: larval morphology of *Xenohyla truncata* (Anura: Hylidae: Dendropsophini) and its implication to the evolution of Dendropsophini tadpoles. Zoomorphology 142:111–126

[CR23] Dias PHS, Vera Candioti F, Wassersug R, Lukas P, Targino M, Glos J, Haas A (2024) Stranger things: on the novel buccopharyngeal anatomy and functional morphology of ‘sand-eating’Malagasy tadpoles (Anura: Mantellidae: *Mantidactylus*). Zool J Linn Soc 202:zlae127

[CR24] Dubeux MJM, Do Nascimento FAC, Dias PHS (2023) Larval morphology of *Frostius pernambucensis* (Anura): contribution of larval characters for the systematics of the family Bufonidae and evolution of endotrophic tadpoles. Zoomorphology 143:59–187

[CR25] Duellman WE, Marion AB, Hedges B (2016) Phylogenetics, classification, and biogeography of the treefrogs (Amphibia: Anura: Arborana). Zootaxa 4104:1–109 27394762 10.11646/zootaxa.4104.1.1

[CR26] Echeverría DD (1997) Microanatomy of the buccal apparatus and oral cavity of *Hyla minuta* Peters, 1872 (Anura, Hylidae), with data on feeding habits. Alytes 15:26–36

[CR27] Faivovich J, Haddad CFB, Garcia PCA, Frost DR, Campbell JA, Wheeler WC (2005) Systematic review of the frog family Hylidae, with special reference to Hylinae: phylogenetic analysis and taxonomic revision. Bull Am Mus Nat Hist 294:1–240

[CR28] Faivovich J, Pereyra MO, Luna MC, Hertz A, Blotto BL, Vásquez-Almazán CR, Haddad CFB (2018) On the monophyly and relationships of several genera of Hylini (Anura: Hylidae: Hylinae), with comments on recent taxonomy changes in hylids. S Am J Herpetol 13:1–32

[CR29] Fitch WM (1971) Toward defining the course of evolution: minimum change for a specific tree topology. Syst Biol 20:406–416

[CR30] Frost DR, Grant T, Faivovich J, Bain RH, Haas A, Haddad CFB, De Sá RO, Channing A, Wilkinson M, Donnellan SC, Raxworthy CJ, Campbell JA, Blotto BL, Moler P, Drewes RC, Nussbaum RA, Lynch JD, Green DM, Wheeler WC (2006) The amphibian tree of life. Bull Am Mus Nat Hist 297:1–291

[CR31] Goloboff PA, Morales ME (2023) TNT version 1.6, with a graphical interface for MacOS and Linux, including new routines in parallel. Cladistics 39:144–153 36682054 10.1111/cla.12524

[CR32] Gomes FBR, Provete DB, Martins IA (2010) The tadpole of *Physalaemus jordanensis* Bokermann, 1967 (Anura, Leiuperidae) from Campos do Jordão, Serra da Mantiqueira, Southeastern Brazil. Zootaxa 2327:65–68

[CR33] Gosner KL (1960) A simplified table for staging anurans embryos and larvae with notes on identifications. Herpetologica 16:183–190

[CR34] Grant T, Kluge AG (2004) Transformation series as an ideographic character concept. Cladistics 20:23–31 34892973 10.1111/j.1096-0031.2004.00003.x

[CR35] Hennig W (1966) Phylogenetic systematics. University of Illinois Press, Urbana, p 263

[CR36] Jetz W, Pyron RA (2018) The interplay of past diversification and evolutionary isolation with present imperilment across the amphibian tree of life. Nat Ecol Evol 2:850–858 29581588 10.1038/s41559-018-0515-5

[CR37] Kaplan M, Ramírez-Bautista A (1996) Description of the tadpole of *Hyla plicata* with comments on the taxonomic value of the larval internal oral morphology. J Herpetol 30:530–533

[CR38] Kaplan M, Ruíz-Carranza PM (1997) Two new species of *Hyla* from the Andes of central Colombia and their relationships to the other small Andean *Hyla*. J Herpetol 31:230–244

[CR39] Kenny JS (1969a) Feeding mechanisms in anuran larvae. J Zool 157:225–246

[CR40] Kenny JS (1969b) Pharyngeal mucous secreting epithelia of anuran larvae. Acta Zool 50:143–153

[CR41] Kolenc F, Borteiro C, Alcalde L, Baldo JD, Cardozo D, Faivovich, J (2008) Comparative larval morphology of eight species of *Hypsiboas* Wagler (Amphibia, Anura, Hylidae) from Argentina and Uruguay, with a review of the larvae of this genus. Zootaxa 1927:1–66

[CR42] Maddison W, Maddison D (2021) Mesquite: a modular system for evolutionary analysis. Version 3.70 (August 2021). Accessible at http://mesquiteproject.org. Accessed 31 Aug 2021

[CR43] Marcondes BC, Dias PHS, Araújo RB, de Lima GCF, Cuervo‐Santos C, Oswald CB, Pezzuti TL (2025) Suction feeding in *Dendropsophus cerradensis* tadpoles: new behavioral observations and morphological traits in a member of the D. *microcephalus* group (Anura, Hylidae). J Morphol 286:e70050 10.1002/jmor.70050 PMC12057304 40331568

[CR44] Nunes I, Fusinatto LA, Cruz CAG (2007) The tadpole and advertisement call of *Sphaenorhynchus palustris* Bokermann, 1966 (Amphibia, Anura, Hylidae). S Am J Herpetol 2:123–128

[CR45] Orrico VGD, Grant T, Faivovich J, Rivera-Correa M, Rada MA, Lyra ML, Cassini CS, Valdujo PH, Schargel WE, Machado DJ, Wheeler WC, Barrio‐Amorós C, Loebmann D, Moravec J, Zina J, Solé M, Sturaro MJ, Peloso PLV, Suarez P, Haddad CFB (2021) The phylogeny of Dendropsophini (Anura: Hylidae: Hylinae). Cladistics 37:73–105 34478175 10.1111/cla.12429

[CR46] Portik DM, Streicher JW, Wiens JJ (2023) Frog phylogeny: a time-calibrated, species-level tree based on hundreds of loci and 5,242 species. Mol Phylogenet Evol 188:107907 37633542 10.1016/j.ympev.2023.107907

[CR47] Provete DB, Garey MV, Picheli KO, Mello CM, Da Silva GD, Conte EC, Rossa-Feres DC (2021) The tadpoles and advertisement call of *Trachycephalus imitatrix* and *T. dibernardoi* (Anura: Hylidae). J Herpetol 55:237–252

[CR48] Pyron A, Wiens JJ (2011) A large-scale phylogeny of Amphibia including over 2800 species, and a revised classification of extant frogs, salamanders, and caecilians. Mol Phylogenet Evol 61:543–583 21723399 10.1016/j.ympev.2011.06.012

[CR49] Roberto IJ, Araujo-Vieira K, Carvalho-e-Silva SP, Ávila RW (2017) A new species of *Sphaenorhynchus* (Anura: Hylidae) from northeastern Brazil. Herpetologica 73:148–161

[CR50] Sabaj MH (2022) Codes for natural history collections in ichthyology and herpetology (online supplement). Version 9.0 (14 February 2022). American Society of Ichthyologists and Herpetologists, Washington. Available at https://asih.org. Accessed 14 Feb 2022

[CR51] Sandoval MT (2000) Microanatomía del disco oral y cavidad bucofaringea de la larva *Scinax acuminatus* (Anura: Hylidae). Facena 16:93–102

[CR52] Silva Neto EM, Araujo-Vieira K, Dias IR, Batista CS, Pareja-Mejía D, Solé M, de Mira-Mendes CV (2022) Redescription of the tadpoles of *Gabohyla pauloalvini* and *Sphaenorhynchus prasinus* (Hylidae: Sphaenorhynchini). J Herpetol 56:422–433

[CR53] Suárez-Mayorga AM, Lynch JD (2001) Los renacuajos colombianos de *Sphaenorhynchus* (Hylidae), descripciones, anotaciones sistemáticas y ecológicas. Rev Acad Colomb Cienc Exactas Fis Nat 25:411–420

[CR54] Tschudi JJv (1838) Classification der Batrachier mit Berücksichtigung der fossilen Thiere dieser Abtheilung der Reptilien. Neuchâtel: Petitpierre 1838:1–111

[CR55] Vera Candioti MF (2007) Anatomy of anuran tadpoles from lentic water bodies systematic relevance and correlation with feeding habits. Zootaxa 1600:1–175

[CR56] Vera Candioti MF, Dias PHS, Rowley JJ, Hertwig S, Haas A, Altig R (2021) Anatomical features of the phytotelma dwelling, egg-eating, fanged tadpoles of *Rhacophorus vampyrus* (anura: rhacophoridae). J Morphol 282:769–778 33713040 10.1002/jmor.21348

[CR57] Viertel B (1982) The oral cavities of central European anuran larvae (Amphibia): morphology, ontogenesis and generic diagnosis. Amphibia-Reptilia 4:327–360

[CR58] Wassersug RJ (1976) Oral morphology of anuran larvae: terminology and general descriptions. Occas Pap Mus Nat Hist Univ Kans 48:1–23

[CR59] Wassersug RJ (1980) Internal oral features of larvae from eight anuran families: functional, systematic, evolutionary, and ecological consideration. Misc Publ Mus Nat Hist Univ Kansas 68:1–148

[CR60] Wassersug RJ, Heyer WR (1988) A survey of internal oral features of Leptodactyloid larvae (Amphibia: Anura). Smith Contr Zool 457:1–99

