# Supplementary material for: Comparative buccopharyngeal morphology of tadpoles of Sphaenorhynchini (Anura: Hylidae: Hylinae)
Source: Naturwissenschaften. 2025 Aug 21;112(5):62. doi: 10.1007/s00114-025-02009-8 (PMC12370870; doi:10.1007/s00114-025-02009-8)
Supplement: Supplementary file 1 — (DOCX 22.4 KB) [file 114_2025_2009_MOESM1_ESM.docx]

**Table S1** List of the sources of character state scorings in the phenotypic data matrix provided in Appendix S1. Data current through April 2025

| **Taxon** | **Reference** |
| --- | --- |
| *Julianus uruguayus* | Alcalde et al. (2011) |
| *Ololygon berthae* | Alcalde et al. (2011) |
| *Trachycephalus atlas* | Barreto et al. (2015) |
| *Boana leptolineata* | Both et al. (2007) |
| *Hyla chinensis* | Chou and Lin (1997) |
| *Ololygon catharinae* | Conte et al. (2007) |
| *Boana geographica* | D’Heursel and de Sá (1999) |
| *Boana semilineata* | D’Heursel and de Sá (1999) |
| *Aplastodiscus albofrenatus* | D’Heursel and Haddad (2007) |
| *Aplastodiscus albosignatus* | D’Heursel and Haddad (2007) |
| *Aplastodiscus cochranae* | D’Heursel and Haddad (2007) |
| *Aplastodiscus eugenioi* | D’Heursel and Haddad (2007) |
| *Aplastodiscus perviridis* | D’Heursel and Haddad (2007) |
| *Boana albomarginata* | D’Heursel and Haddad (2007) |
| *Boana cinerascens* | D’Heursel and Haddad (2007) |
| *Boana joaquini* | D’Heursel and Haddad (2007) |
| *Bokermannohyla alvarengai* | D’Heursel and Haddad (2007) |
| *Bokermannohyla luctuosa* | D’Heursel and Haddad (2007) |
| *Bokermannohyla itapoty* | D’Heursel and Haddad (2007) |
| *Bokermannohyla nanuzae* | D’Heursel and Haddad (2007) |
| *Bokermannohyla saxicola* | D’Heursel and Haddad (2007) |
| *Dendropsophus minutus* | Echeverría (1997) |
| *Litoria caerulea* | Dias (2023) |
| *Litoria rubella* | Dias (2023) |
| *Phasmahyla cruzi* | Dias et al. (2018) |
| *Phasmahyla guttata* | Dias et al. (2018) |
| *Dendropsophus decipiens* | Dias et al. (2019) |
| *Xenohyla truncata* | Dias et al. (2023) |
| *Bokermannohyla ahenea* | Garey et al. (2020) |
| *Trachycephalus resinifictrix* | Grillitsch (1992) |
| *Hyla plicata* | Kaplan and Ramírez-Bautista (1996) |
| *Boana caingua* | Kolenc et al. (2008) |
| *Boana cordobae* | Kolenc et al. (2008) |
| *Boana faber* | Kolenc et al. (2008) |
| *Boana pulchella* | Kolenc et al. (2008) |
| *Boana punctata* | Kolenc et al. (2008) |
| *Boana raniceps* | Kolenc et al. (2008) |
| *Boana riojana* | Kolenc et al. (2008) |
| *Osteopilus ocellatus* | Lannoo et al. (1987) |
| *Osteopilus septentrionalis* | Lannoo et al. (1987) |
| *Boana bandeirantes* | Luna-Dias et al. (2019) |
| *Boana polytaenia* | Luna-Dias et al. (2019) |
| *Phyllodytes wuchereri* | Magalhães et al. (2015a) |
| *Bokermannohyla oxente* | Magalhães et al. (2015b) |
| *Dendropsophus cerradensis* | Marcondes et al. (2025) |
| *Boana jaguariaivensis* | Mello et al. (2021) |
| *Bokermannohyla circundata* | Mongin and Carvalho-e-Silva (2013) |
| *Boana atlantica* | Nascimento et al. (2009) |
| *Phyllomedusa ayeaye* | Pezzuti et al. (2009) |
| *Trachycephalus dibernadoi* | Provete et al. (2021) |
| *Trachycephalus imitatrix* | Provete et al. (2021) |
| *Corythomantis greeningi* | Oliveira et al. (2017) |
| *Boana crepitans* | Oliveira et al. (2021) |
| *Scinax acuminatus* | Sandoval (2000) |
| *Phyllomedusa azurea* | Santos et al. (2018) |
| *Phyllomedusa oreades* | Santos et al. (2018) |
| *Scinax constrictus* | Santos et al. (2019) |
| *Dendropsophus nanus* | Vera Candioti et al. (2004) Vera Candioti (2007) |
| *Scinax nasicus* | Vera Candioti et al. (2004) Vera Candioti (2007) |
| *Dendropsophus microcephalus* | Vera Candioti (2007) |
| *Boana rosenbergi* | Vera Candioti (2007) |
| *Lysapsus limellum* | Vera Candioti (2007) |
| *Phyllomedusa hypochondrialis* | Vera Candioti (2007) |
| *Phyllomedusa sauvagii* | Vera Candioti (2007) |
| *Pseudis paradoxa* | Vera Candioti (2007) |
| *Scinax boulengeri* | Vera Candioti (2007) |
| *Phyllodytes brevirostris* | Vieira et al. (2009) |
| *Hyla arborea* | Viertel (1982) |
| *Pseudacris regilla* | Wassersug (1976a) |
| *Acris crepitans* | Wassersug (1980) |
| *Agalychnis callidryas* | Wassersug (1980) |
| *Boana rufitela* | Wassersug (1980) |
| *Dendropsophus ebraccatus* | Wassersug (1980) |
| *Dendropsophus phlebodes* | Wassersug (1980) |
| *Dendropsophus sarayacuensis* | Wassersug (1980) |
| *Hyla femoralis* | Wassersug (1980) |
| *Megastomatohyla mixe* | Wassersug (1980) |
| *Ptychohyla leonhardschultzei* | Wassersug (1980) |
| *Smilisca sordida* | Wassersug (1980) |
| *Triprion spinosus* | Wassersug (1980) |

**References not included in the main text**

Barreto GS, Ramos JC, Napoli MF. Garda AA, Juncá FA (2015) External morphology and oral cavity of the tadpole of *Trachycephalus atlas* Bokermann, 1966 (Amphibia, Anura, Hylidae). Zootaxa 3980:597–600.

Both C, Kwet A, Solé M (2007) The tadpole of *Hypsiboas leptolineatus* (Braun and Braun, 1977), a species in the *Hypsiboas polytaenius* clade (Anura; Hylidae). Braz J Biol 67:309–312.

Chou WH, Lin JY (1997) Tadpoles of Taiwan. Nat Mus Nat Sci,No. 7. iv+1−98.

Dias PHS, Mongin-Aquino M, Candioti FV, Carvalho AMPT, Baêta D (2018b) Internal larval morphology of two species of shining leaf frogs (Anura: Phyllomedusidae: *Phasmahyla*). S Am J Herpetol 13:44–53.

Garey MV, Provete DB, Ouchi-Melo LS, Haddad CFB, Rossa-Feres DDC (2020) The larva and advertisement call of *Bokermannohyla ahenea* (Anura: Hylidae). S Am J Herpetol 17:1−16.

Grillitsch B (1992) Notes on the tadpole of *Phrynohyas resinifictrix* (Goeldi, 1907). Buccopharyngeal and external morphology of a tree hole dwelling larva (Anura, Hylidae). Herpetozoa 5:51–66.

Luna-Dias C, Ruggeri J, Carvalho-e-Silva SP (2019) Oral cavity of the tadpoles of *Boana bandeirantes* (Caramaschi and Cruz, 2013) and *B. polytaenia* (Cope, 1870), with a new state record for *B. bandeirantes* (Amphibia, Anura, Hylidae). Herpetol Notes 12:537–542.

Magalhães FM, Juncá FA, Garda AA. (2015a) Tadpole and vocalisations of *Phyllodytes wuchereri* (Anura: Hylidae) from Bahia, Brazil. Salamandra, 51(2).

Magalhães FM, Mercês EA, Santana DJ, Juncá FA, Napoli MF, Garda AA (2015b) The tadpole of *Bokermannohyla flavopicta* Leite, Pezzuti and Garcia, 2012 and oral cavity anatomy of the tadpole of *B. oxente* Lugli and Haddad, 2006 (Anura: Hylidae). S Am J Herpetol 10:211–218.

Mello CM, Gonçalves DDS, Rossa-Feres DDC, Conte CE (2021) The tadpole of *Boana jaguariaivensis* (Caramaschi, Cruz & Segalla, 2010)(Anura: Hylidae): external and internal oral cavity morphology. Zootaxa 5047:495–500.

Mongin MM, Carvalho-e-Silva AMPT (2013) Descrição da morfologia oral interna, ontogenia eredescrição do girino de *Bokermannohyla circumdata* (Cope, 1870) (Amphibia: Anura: Hylidae). Bol. Mus. Para. Emilio Goeldi 8:133–152.

Nascimento FA, Lima MGD, Skuk GO, de Sá RO (2009) The tadpole of *Hypsiboas atlanticus* (Anura, Hylidae) from northeastern Brazil. Iheringia Sér Zool 99:431–436.

Oliveira MIRR, Weber LN, de Sá RO, Ferreira JS, Libório AEC, Takazone AMG (2017) Chondrocranium and internal oral morphology of the tadpole of *Corythomantis greeningi* (Anura: Hylidae). Phyllomedusa 16:71–80.

Oliveira MIRR, Weber LN, Ferreira JS, Coimbra LAE, Takazone AMG, de Sá RO (2021) Larval chondrocranial and internal oral morphology of the neotropical treefrog *Boana crepitans* (Wied‐Neuwied, 1824; Amphibia, Anura, Hylidae). J Morphol 282:1274–1281.

Santos DL, De Morais AR, Signorelli L, Bastos RP, Feio R, Nomura F (2018) Description of the tadpole of *Phyllomedusa azurea* from the Brazilian Cerrado, with a description of the internal oral morphology of *Phyllomedusa oreades*. Herpetologica 74:50–57.

Santos DL, Gambale PG, Bastos RP, Nomura F (2019) The tadpole of *Scinax constrictus* Lima, Bastos & Giaretta, 2004 (Amphibia, Hylidae). J Herpetol 53:62–67.

Vera Candioti MF, Lavilla EO, Echeverría DD (2004) Feeding mechanisms in two treefrogs, *Hyla nana* and *Scinax nasicus* (Anura: Hylidae). J Morphol 261:206–224.

Vieira WL, Santana GG, Santos SCNC, Alves RRN, Pereira-Filho GA (2009) Description of the tadpoles of *Phyllodytes brevirostris* (Anura: Hylidae). Zootaxa 2119:66–68.
